# Hospital-Based Quasi-Experimental Study on Hydroxychloroquine Pre-Exposure Prophylaxis for COVID-19 in Healthcare Providers with Its Potential Side-Effects

**DOI:** 10.3390/life12122047

**Published:** 2022-12-07

**Authors:** Lubaba Shahrin, Mustafa Mahfuz, Md. Waliur Rahman, Md. Rezaul Hossain, Afsana Mim Khandaker, Md. Ashraful Alam, Din M. M. F. Osmany, Md. Munirul Islam, Mohammod Jobayer Chisti, Chaudhury Meshkat Ahmed, Tahmeed Ahmed

**Affiliations:** 1 Dhaka Hospital, International Centre for Diarrheal Disease Research, Bangladesh (icddr, b), Dhaka 1212, Bangladesh; 2 Nutrition and Clinical Services Division, International Centre for Diarrheal Disease Research, Bangladesh (icddr, b), Dhaka 1212, Bangladesh; 3 Department of Epidemiology, University of Washington, Seattle, WA 98195, USA; 4 Department of Nutrition, University of Nevada, Reno, NV 89557, USA; 5 Department of Cardiology, Bangabandhu Sheikh Mujib Medical University, Dhaka 1000, Bangladesh

**Keywords:** hydroxychloroquine, COVID-19, preexposure prophylaxis, corrected QT interval, healthcare workers

## Abstract

Considering that it has been more than 24 months since SARS-CoV-2 emerged, it is crucial to identify measures that prevent and control pathogen transmission in workplace settings. Our aim was to report results of a hospital-based program that delivered hydroxychloroquine (HCQ) tablets as COVID-19 prophylaxis to the frontline healthcare workers (HCW)s who cared for COVID-19 patients and to evaluate the efficacy of HCQ. Setting and participants: Quasi-experimental, controlled, single-center study. The included participants were doctors, nurses, health workers, cleaning staff, and non-healthcare supportive staff. The main outcome was contracting COVID-19 anytime during the period of taking the prophylaxis, confirmed by RT-PCR. A total of 336 participants, without any clinical evidence of COVID-19 and without any known contact with family members, were included in the trial; 230 were assigned to HCQ and 106 declined to take any drug. Results: Among the participants, 43 (18.7%) in the HCQ group and 11 (10.4%) participants in the control group developed COVID-19. For the evaluation of side effects, we evaluated 12-lead ECGs of both groups at the baseline and after 4 weeks to monitor QTc interval. A total of 91% (198 of 217) participants in the prophylaxis group and 92% (11 of 12) in the control group had a QTc < 45o msec, which is within normal limits. Conclusions: Although the number of symptomatic infections in health personnel was lower in the control group, the difference was not statistically significant. However, in the absence of any effective pre-exposure prophylaxis medicine for COVID-19, practicing proper infection prevention and control (IPC) and vaccination is the only way forward.

## 1. Background

Severe Acute Respiratory Syndrome Coronavirus-2 (SARS-CoV-2) or Coronavirus disease (COVID-19) has caused many deaths and morbidities around the world and is considered to be a matter of serious public health concern [1,2,3,4,5,6]. SARS-CoV-2 is exceedingly contagious (the reproduction number, “R naught” (*R*_0_) ranges from 2.24 to 3.58 worldwide) and it notably affected front-line healthcare workers (HCWs) who treated COVID-19 patients [7,8,9]. As of the latest report, 37 million cases of COVID-19 among health workers from 183 countries and regions were reported to the World Health Organization (WHO), a figure that represents 36% of the total cases [10]. Although the ongoing development of SARS-CoV-2 vaccines is regarded as the most promising preventive strategy, an urgent alternative until then would be to re-purpose already existing pharmacological agents as a prophylaxis for high-risk communities, such as the HCWs [11].

Hydroxychloroquine (HCQ, an antimalarial agent) has been shown to have some merits in preventing SARS-CoV-2 in vitro, and different groups around the globe have advocated its use as a prophylactic drug for COVID-19 [12,13,14]. Several retrospective studies have demonstrated some advantages of HCQ for COVID-19 treatment and other clinical studies have reported mixed findings [15]. Recently, Boulware et al. published the result of their “pragmatic” randomized trial, which showed that the use of HCQ as a post-exposure prophylaxis did not prevent infection, despite a number of acknowledged methodological limitations [11,16]. In order to verify the effectiveness and safety of HCQ in a high-risk HCW population as a pre-exposure prophylaxis, a triple-blinded, randomized clinical trial already began in Mexico (NCT04318015), but the trial stopped prematurely [17]. Beyond this, a systematic review reveals very weak and contradictory evidence on the benefits and drawbacks of using HCQ to treat or prevent COVID-19 to date [15].

Here, we report results of a hospital-based program that delivered hydroxychloroquine tablets as COVID-19 prophylaxis to the frontline HCWs who cared for COVID-19 patients. Our report also addresses the dose–response relationship, cardio-toxicity, and ocular toxicity, along with other side effects of HCQ prophylaxis.

## 2. Methods

### 2.1. Setting and Study Type

The study was conducted in the Dhaka hospital of the International Centre for Diarrheal Disease Research, Bangladesh (icddr, b), which is the largest diarrhea hospital in the world. During the pandemic, to deal with unidentified numbers of patients with asymptomatic COVID-19, we developed a TRIAGE system, recommended by the World Health Organization. A part of the hospital was converted to treat COVID-19 patients. For the healthcare workers who are at risk while working in the COVID-19 makeshift facility, HCQ tablets were offered as a pre-exposure prophylaxis service in the hospital. Informed consent was collected from the staff before providing the medicine.

### 2.2. Participants

The target populations were doctors, nurses, health workers, cleaning staff, and non-healthcare supportive staff (kitchen staff, dietician, and laundry attendant) who were involved in direct patient care. They had a potential risk of contracting suspected or confirmed COVID-19 patients in the hospital triage, in-patient department, and intensive care units. Clinically asymptomatic staff members who were willing to comply with the treatment protocol were included to the intervention arm. Staff members who were known cases of myocardial infarction or ischemic heart disease, pregnant women, anyone hypersensitive to hydroxychloroquine or 4-aminoquinoline compounds, those with a baseline ECG showing QTc over 480 msec, or staff who were positive for a RT-PCR test or have a history of any cardiac surgeries were excluded from the intervention. Staff members who declined taking HCQ tablets as prophylaxis were counted as the controls for this analysis.

### 2.3. Ethical Approval

No ethical approval from the institutional review board was taken during the service due to the time constraint and consideration of immediate pre-exposure issues of the staff working in the hospital. After careful consideration of the existing health status of the individuals, informed consent was taken prior to enrolment. Afterward, the data were used to evaluate the findings and consent was obtained from the staff to use the de-identified data for publication. The research was approved by the Research Review Committee of icddr, b (PR-20014; approval date 5 April 2022).

### 2.4. Randomization and Blinding

Randomization could not be possible, as this was a voluntary service for the staff. The control group comprised those who refused to take the medicine, and the intervention arm comprised the remainder. Blinding was also not considered for this service due to following infection prevention measures adequately.

### 2.5. Intervention

We offered oral hydroxychloroquine (400 mg) tablets to the HCWs as prophylaxis. We initiated the activity on 25 March 2020, and continued until 12 July 2020. The drug was dispensed as ‘Directly Observed Treatment (DOT)’ twice a day on Day 1, followed by once weekly for the next 7 weeks, to be taken with meals. A 20 mg zinc tablet was also given daily for 10 days.

### 2.6. Recruitment and Discontinuation Procedure

**Figure 1:**
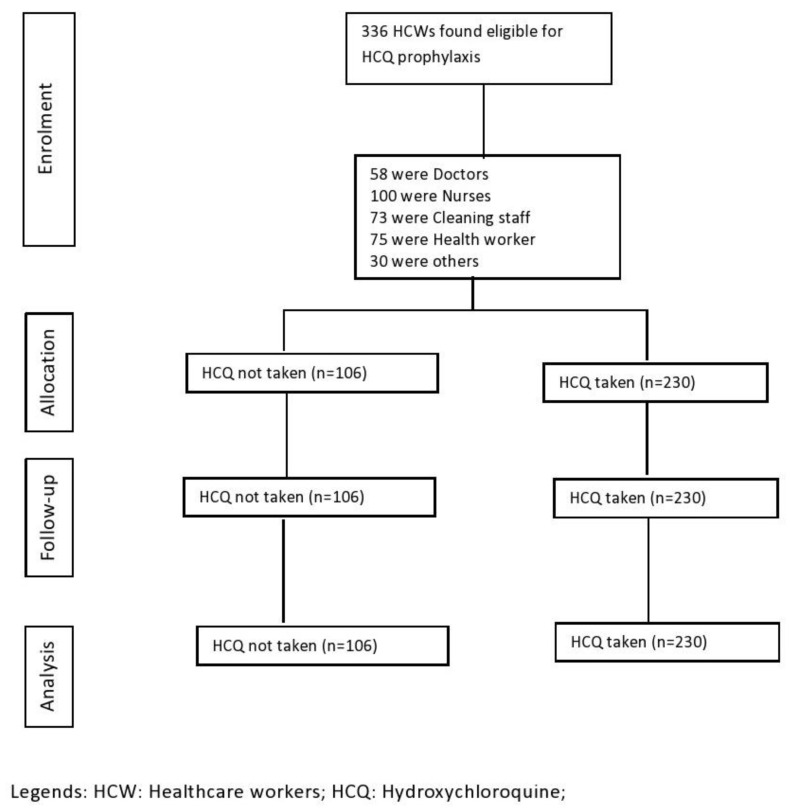
CONSORT flow diagram of quasi-experimental study of HCQ use in healthcare providers.

The HCWs were contacted over the telephone and were informed of the service. Once the staff agreed to participate, they were given an appointment to have an electrocardiogram (ECG) performed. The ECG reports were examined by the on-duty physician, and if no abnormalities were detected, the staff was assigned the medicine. In doubtful cases, the ECG tracings were shown to another senior physician for a final decision. HCQ drugs were dispensed from the pharmacy upon presenting the normal ECG tracing findings and satisfying the absence of exclusion criteria. Every patient who received the drug by DOT from the pharmacy were given two phone numbers to report any adverse reactions or side effects after taking the HCQ. The descriptions of side effects from those who called were documented in the database according to their HCQ dose count. At the end of the dose count, information regarding side effects was collected over the telephone. The overall schema of recruitment is detailed in Figure 1. The participation was voluntary and they had the option to discontinue the drug at any time. Moreover, while receiving HCQ prophylaxis, if any of the participants developed COVID-like symptoms, the were tested by RT-PCR technique. If the result came out to be positive, they were withdrawn from the prophylaxis therapy and treated according to the guidelines. Individuals with a QTc interval >480 msec or Torsade score >6 were also advised to discontinue the drug.

### 2.7. Evaluation of Toxicity

We evaluated the participants for possible development of cardiac, ocular, and other toxicities. Considering the fact that hydroxychloroquine might cause QT prolongation and associated cardiac events, regular monitoring for cardiac toxicity was conducted by a sequence of ECGs. The first 35 ECG tracings were read and cross-examined by three physicians independently. Afterward, the remainder of the ECG tracings was read by a physician, and in doubtful cases, consultations were sought from senior cardiologists. The decision schema on baseline ECG can be found in Supplementary Table S1. Risk factors were assessed by Torsade’s de Pointes (TdP) scoring system developed and validated by Tisdale et al. for the prediction of drug-associated QT prolongation among cardiac-care-unit-hospitalized patients. A score ≤6 predicts low risk and ≥6 predicts medium risk of developing QT prolongation For evaluating the score before the treatment, a few questions, including a detailed drug history, were asked and the Tisdale risk factor assessment scores were calculated (Supplementary Table S2). Each participant also had a follow-up ECG after the completion of 4 weeks (during the 5th week drug) of drug intake, and the recording were compared with the baseline ECG results to monitor any changes. Participants with a QTc < 450 msec had a single repeat ECG at the end of 4th week, and the remainder of the participants with QTc > 450 msec had repeat ECGs weekly, with the last ECG being performed at the end of the 7th week (usually 49 days after baseline).

To evaluate ocular toxicity, ophthalmological tests were conducted: (i) clinical ophthalmological examination, (ii) fundus photo, (iii) visual field test, and (iv) optical coherence tomography (for selected cases based on the ophthalmologist’s decision). If an individual was more than 40 years of age or suffering from diabetes mellitus, they were offered an eye examination, irrespective of hydroxychloroquine consumption. Once listed, individuals were contacted over the phone. The participation in the eye check-up was voluntary and optional. Upon agreement, they were then scheduled on a first-come-first-served basis, starting with the prophylaxis group, as they were readily available for prescription at the hospital. Eye check-up and examination were carried out in a separate privately-owned eye hospital approximately three kilometers away from the center, and one particular senior eye consultant was appointed for conducting the eye examinations and for providing the reports. The eye consultant first recorded any known pre-existing eye pathology and then carried out a general eye examination on every individual, including visual acuity, intraocular pressure, and optometry. Then, a 10-2 Humphrey visual field testing (perimetry) was carried out on all selected individuals, followed by fundus auto-fluorescence imaging (fundus photo) after pupillary dilation. An optical coherence tomography (OCT) was carried out on a subset of individuals whose visual field test or fundus photograph showed eye changes. Performance of OCT was completely at the discretion of the eye consultant.

### 2.8. Patient and Public Involvement

Staff engaged in COVID-19 care (study participants) were informed of the individual results of the baseline examination as well as on the primary and secondary outcomes of the study in the form of individual consultation with a research team member. Study participants were not involved in the development of the research question or study design.

### 2.9. Statistical Analysis

We reported categorical variables as number (%) and continuous variables as median (IQR) to describe the demographic characteristics of the participants. The data were analyzed by statistical software Stata 15.1. (StataCorp LLC, College Station, TX, USA). We compared baseline characteristics, side effects, and other descriptive features of intervention group with the control group using chi-square tests and Mann–Whitney *U* tests, as appropriate. Multivariable logistic regression models were fit to measure the effect of prophylaxis and other covariates on the outcome. Initially we fit bivariate logistic regression models to 5% level of significance, and the variables with *p*-values <0.20 in the bivariate analysis were included in the multivariable model. The associations were expressed as the adjusted odds ratios and 95% confidence intervals. A *p*-value of <0.05 was considered statistically significant for the multivariable logistic regression model.

To explore the association of hydroxychloroquine doses and the time to develop the disease, survival analysis with Cox proportional hazard model was conducted. Bivariate associations between each independent variable and the time to develop the disease were measured using unadjusted Cox proportional hazards models, and variables associated with time to develop COVID-19 at the level of *p*-value <0.20 were included in the multivariable model. For the multivariable model, statistical significance was defined at a *p*-value less than 0.05.

## 3. Results

A total of 230 healthcare workers worked at the Dhaka hospital of icddr, b received HCQ and 106 healthcare workers did not receive it. The demographic and clinical characteristics of the participants are detailed in Table 1. The median age was 34 years (interquartile range, IQR 25–43 years) in the prophylaxis group and 40 years (IQR, 29–55) in the control group. Regarding sex distribution, it was 40% male in both the prophylaxis group and control group. A total of 16% of the participants reported to have hypertension in the prophylaxis group and 23% in the control group. The median days of working was 23 days (IQR, 16–41) in the prophylaxis group and 9 days (IQR, 0–16) in the control group. One-third of the participants (32%) of the prophylaxis group were nurses.

### 3.1. Primary Outcome

Overall, a total of 54 (16%) HCWs developed COVID-19 (confirmed by RT-PCR analysis) between 31 March 2020, and 12 July 2020. The incidence of COVID-19 did not differ significantly between the prophylaxis (18.7%) and control groups (10.4%) (*p* = 0.054).

The unadjusted (OR: 1.99, 95% CI: 0.98, 4.03; *p* = 0.057) and adjusted (OR: 2.09, 95% CI: 0.90, 4.85; *p* = 0.088) analyses suggest that prophylaxis group participants had a higher risk of contracting COVID-19 than the control group, but the differences were not statistically significant. In a bivariate analysis, we identified employment type, hypertension, and total days of work as having statistically significant association with contracting COVID-19 (Table 2), whereas age, gender, bronchial asthma/COPD, and smoking status were not statistically significant (*p* > 0.05). In the multivariable model, employment type of “Health worker” (OR: 3.43, 95% CI: 1.09, 10.82; *p* = 0.036) and “Other” (OR: 6.18, 95% CI: 1.74, 21.97; *p* = 0.005) were significantly associated with COVID-19 positivity (Table 2).

### 3.2. Subgroup Analysis-1

In this subgroup analysis, we included the participants who worked for at least for 16 days and excluded those participants who were affected with COVID-19 within 14 days (highest days of incubation period) of receiving the 1st dose. With this above-mentioned criterion, a total of 172 healthcare workers received HCQ and 30 healthcare workers did not. The unadjusted (OR: 0.77, 95% CI: 0.27, 2.22; *p* = 0.631) and adjusted (OR: 0.92, 95% CI: 0.28, 3.01; *p* = 0.894) analyses suggest that the prophylaxis group participants had lower risk of being COVID-19 positive than the control group, but this was not statistically significant (Table 3).

### 3.3. Subgroup Analysis-2

In this subgroup analysis, we excluded only the participants who were affected with coronavirus within 14 days of receiving the 1st dose. On the basis of the criteria, we included data from a total of 225 healthcare workers who received HCQ and 106 healthcare workers did not receive HCQ. The unadjusted (OR: 1.75, 95% CI: 0.85, 3.59; *p* = 0.123) and adjusted (OR: 1.74, 95% CI: 0.74, 4.09; *p* = 0.207) analyses suggest that the prophylaxis group participants had higher risk of contracting COVID-19 than the control group, but this is not statistically significant (Table 4).

### 3.4. Dose–Response Analysis

In this analysis, we included all the participants who received HCQ. The Cox proportional hazard regression model showed that “total number of doses” (Adjusted HR: 0.85, 95% CI: 0.72, 0.99; *p* = 0.042) was significantly associated with time to develop the disease (Table 5).

### 3.5. Evaluation of Toxicity

Most of the participants did not report any side effects after receiving HCQ (Table 6). Only 9% of the participant (21 of 229) reported symptoms during the medication period, including palpitation (0.4%), neurological problems (2.2%), skin problems (1.3%), visual problems (0.4%), weakness (0.4%), fever (0.4%), GI problems (3.1%), and anxiety (0.4%).

ECG was performed on 217 participants in the prophylaxis group, before they started receiving the medication, and 12 in the control group. Most of the participants’ corrected QT interval was <450 msec, which was 91% (198 of 217) in the prophylaxis group and 92% (11 of 12) in the control group. A total of 8% (17 of 217) participants had a corrected QT interval between 450 to 480 msec in the prophylaxis group and 8% (1 of 12) in the control group. Only two participants in the prophylaxis group had a corrected QT interval of >480 msec. A second ECG was performed on 105 participants in the prophylaxis group during HCQ intervention. ECG reads showed that 94.3% participants had a corrected QT interval of <450 msec, 5.7% had a corrected QT interval between 450 to 480 msec, and only two participants in the prophylaxis group had a QT interval of >480 msec (Table 7).

A total of 73 (32%) individuals in the prophylaxis group and 45 (42%) in the control group were initially listed for eye examination based on their age or existence of diabetes mellitus (Table 8). Of these, 46 (63%) participants in the prophylaxis group and 7 (16%) in the control group completed the eye check-up (Table 8). On average, eye examination was performed after four (sd = 1.7) doses of hydroxychloroquine already taken. Only 2 in the control group and 15 in the prophylaxis group required an optical coherence tomography test. Most of the test parameters identified during the tests were comparable between groups (Table 8). Interestingly, during the visual field analysis, more participants in the prophylaxis group (23 (50%)) presented with unilateral or bilateral scotoma, either centrally or peripherally, than in the control group (2 (29%)). However, the difference was not statistically significant (*p* = 0.3). In addition, none of the variants matched with the classical ring scotoma found in hydroxychloroquine retinopathy. The fundus photography and optical coherence tomography test revealed pre-existing eye conditions, such as cataracts, features of glaucoma, and previous procedures (for example, cataract surgery). However, none of the findings matched with classical features of hydroxychloroquine retinopathy. Three participants were diagnosed with preexisting conditions where hydroxychloroquine use may be avoided. However, only one of them was receiving the prophylaxis, and, by the time the decision of discontinuation was made, already seven out of eight doses were completed. The last dose was withheld.

## 4. Discussion

Although two years have passed since the onset of the pandemic, healthcare workers still remain the most at-risk population for contracting COVID-19. On account of the evolving nature of COVID-19 management facts and precautions, healthcare workers struggled to carry through the protective measures in the workplace. For their protection, no effective prophylaxis for SARS CoV-2 has been discovered to date. The aim of this quasi-experimental study was to determine whether hydroxychloroquine prevents symptomatic COVID-19 infection in healthcare workers.

Chloroquine has been the most effective antimalarial drug discovered in 1934, and is listed as an essential medicine by the World Health Organization [18]. Hydroxychloroquine is the synthesized derivative of chloroquine that has largely replaced chloroquine due to having less adverse effects. Both chloroquine and hydroxychloroquine have shown inhibition of SARS-CoV-2 in cell cultures [19,20,21]. The reason for re-purposing HCQ for SARS-CoV-2 is that HCQ has the property of a remarkably large volume of distribution with extensive pulmonary tissue binding [12,20]. Despite the fact that chloroquine and hydroxychloroquine are shown not to work in the treatment or in post-exposure prophylaxis, it was hypothesized to slow the viral replication in exposed participants [22,23].

A previous study reported the nonbeneficial role of HCQ as a postexposure prophylaxis for COVID-19 if used within 4 days of exposure; however, investigators have described the study design as “pragmatic” [11], due to recruitment of participants through social media and self-reporting of the participants [16]. On the other hand, our study design was quasi-experimental, where we approached the hospital staff who are directly involved in patient care, for example, doctors, nurses, health workers, cleaning staff, and other non-clinical support staff (kitchen staff, dietician, and laundry attendant). The participants were at potential risk of contracting the infection from suspected or confirmed COVID-19 patients. Along with providing HCQ by direct observational therapy, we evaluated them physically for drug-related complications. In addition, we monitored their cardiac and ophthalmologic examinations by expert specialists during the total study period. In summary, our research design should be considered to be more rigorous and systematic compared with the previous mentioned studies.

Our study findings showed that the incidence of COVID-19 did not differ between the participants receiving HCQ prophylaxis and the control group, which is in line with other reported papers on re-purposing of HCQ for pre-exposure prophylaxis [16,24,25,26]. In particular, the major side effects of HCQ that are being skeptically evaluated in other papers [27,28] were better monitored and systematically evaluated in our study by physical evaluation and expert opinion. Furthermore, even after documentation of cardiac toxicity of HCQ in the use of treatment or post-exposure prophylaxis, there was a rational argument for continuing prospective research on HCQ as a prophylaxis for COVID-19 [29].

Although many multinational trials related to HCQ had been placed on hold, the search for alternative drugs for COVID-19 still continues. Ivermectin, which has commonly been used as an antiparasitic agent, has shown promising effects by increasing the likelihood of preventing COVID-19 compared with controls [30,31], although long term follow-up on complications need further study.

There are a few limitations of our study. The first limitation was that the participants did not have any baseline RT-PCR tests performed to confirm their COVID-19 status before beginning the prophylaxis. The second limitation was that the investigator had no control over the participants regarding practicing IPC properly at the workplace or elsewhere. Finally, as the trial was un-blinded, the control group might have taken extra precautions in practicing IPC measures than the prophylaxis group.

Finally, as no pharmacological agents seems to be proved protective as a prophylaxis for COVID-19, we have to reinforce the infection prevention practice and vaccination [32,33], which are still considered as the best practice for the prevention of transmission of SARS-CoV-2.

## Figures and Tables

**Table 1:** Demographic and clinical characteristics of the participants.

Variable	Overall (*n* = 336)	Control (*n* = 106)	Prophylaxis (*n* = 230)	*p*-Value
Age, median (IQR)	35 (26.8, 48.3)	40 (29, 55)	34 (25, 43)	<0.001
Female sex, *n* (%)	203 (60.4%)	64 (60.4%)	139 (60.4%)	0.992
Employment Type, *n* (%)				
Cleaning Staff	73 (21.7%)	10 (9.4%)	63 (27.4%)	<0.001
Doctor	58 (17.3%)	28 (26.2%)	30 (13.1%)	
Health Worker	75 (22.3%)	36 (33.6%)	39 (17.0%)	
Nurse	100 (29.8%)	26 (24.3%)	74 (32.3%)	
Other	30 (8.9%)	6 (5.6%)	24 (10.5%)	
Hypertension, *n*/N (%)	54/307 (17.6%)	20/88 (22.7%)	34/219 (15.5%)	0.134
Bronchial asthma/COPD, *n*/N (%)	29/307 (9.5%)	9/88 (10.2%)	20/219 (9.1%)	0.767
Smoking habit, *n*/N (%)	27/306 (8.8%)	7/88 (7.9%)	20/218 (9.2%)	0.733
Total days of work, median (IQR)	19 (9.8, 33)	9 (0, 16)	23 (16, 41)	<0.001

IQR: Interquartile range; COPD: Chronic Obstructive Pulmonary Disease.

**Table 2:** Bivariate and multivariable relationships between COVID-19 and variables related to demographic and clinical characteristics.

Variable	Unadjusted OR (95% CI)	*p*-Value	Adjusted OR (95% CI)	*p*-Value
Prophylaxis	1.99 (0.98, 4.03)	0.057	2.09 (0.90, 4.85)	0.088
Age (years)	1.01 (0.99, 1.04)	0.332	0.96 (0.92, 1.00)	0.065
Female sex	1.14 (0.62, 2.07)	0.676	1.24 (0.61, 2.52)	0.557
Employment Type				
Doctor	Ref		Ref	
Cleaning staff	0.50 (0.13, 1.87)	0.305	0.65 (0.13, 3.37)	0.611
Health worker	2.94 (1.09, 7.93)	0.033	3.43 (1.09, 10.82)	0.036
Nurse	1.18 (0.42, 3.34)	0.752	0.87 (0.30, 2.57)	0.803
Other	6.63 (2.18, 20.14)	0.001	6.18 (1.74, 21.97)	0.005
Hypertension	2.32 (1.16, 4.63)	0.017	2.07 (0.85, 5.05)	0.109
Bronchial asthma/COPD	2.08 (0.87, 5.00)	0.101	1.43 (0.52, 3.89)	0.486
Smoking habit	0.86 (0.28, 2.60)	0.787		
Total days of work	0.98 (0.96, 0.99)	0.011	0.98 (0.95, 1.01)	0.294

OR: Odds ratio; CI: Confidence interval; COPD: Chronic Obstructive Pulmonary Disease.

**Table 3:** Results of subgroup analysis-1. Unaffected during 14 days incubation period and worked for at least 16 days.

Variable	Unadjusted OR (95% CI)	*p*-Value	Adjusted OR (95% CI)	*p*-Value
Prophylaxis	0.77 (0.27, 2.22)	0.631	0.92 (0.28, 3.01)	0.894
Age (years)	1.02 (0.99, 1.06)	0.229	0.96 (0.91, 1.01)	0.142
Female sex	1.04 (0.46, 2.35)	0.925	1.16 (0.43, 3.15)	0.765
Employment Type				
Doctor	Ref		Ref	
Cleaning staff	0.18 (0.03, 1.05)	0.057	0.14 (0.02, 0.92)	0.041
Health worker	2.91 (0.76, 11.09)	0.117	3.89 (0.74, 20.29)	0.107
Nurse	0.58 (0.16, 2.18)	0.423	0.45 (0.11, 1.79)	0.255
Other	4.13 (0.92, 18.52)	0.064	4.94 (0.87, 28)	0.071
Hypertension	1.83 (0.62, 5.43)	0.276		
Bronchial asthma/COPD	2.98 (0.85, 10.46)	0.089	4.21 (0.88, 20.24)	0.073
Smoking habit	0.6 (0.13, 2.75)	0.512		

IQR: Interquartile range; COPD: Chronic Obstructive Pulmonary Disease.

**Table 4:** Results of subgroup analysis 2: Considering incubation period of 14 days only (Working days not considered).

Variable	Unadjusted OR (95% CI)	*p*-Value	Adjusted OR (95% CI)	*p*-Value
Prophylaxis	1.75 (0.86, 3.59)	0.123	1.74 (0.74, 4.09)	0.207
Age (years)	1.01 (0.99, 1.04)	0.273	0.97 (0.93, 1.01)	0.107
Female sex	1.52 (0.79, 2.91)	0.212	1.7 (0.77, 3.72)	0.187
Employment Type				
Doctor	Ref		Ref	
Cleaning staff	0.25 (0.05, 1.3)	0.099	0.25 (0.04, 1.74)	0.161
HW-A	2.63 (0.96, 7.18)	0.059	3.04 (0.95, 9.75)	0.061
Nurse	1.18 (0.42, 3.34)	0.752	0.84 (0.28, 2.47)	0.748
Other	6.12 (1.99, 18.8)	0.002	6.55 (1.81, 23.68)	0.004
Hypertension	2.19 (1.06, 4.53)	0.034	1.93 (0.77, 4.83)	0.161
Bronchial Asthma/COPD	2.01 (0.8, 5.04)	0.137	1.4 (0.5, 3.96)	0.522
Smoking habit	0.7 (0.2, 2.45)	0.581		
Total days of work	0.98 (0.96, 0.99)	0.015	0.99 (0.97, 1.03)	0.835

IQR: Interquartile range; COPD: Chronic Obstructive Pulmonary Disease.

**Table 5:** Results of Dose–Response Analysis.

Variable	Unadjusted HR (95% CI)	*p*-Value	Adjusted HR (95% CI)	*p*-Value
Total number of doses	0.83 (0.72, 0.96)	0.012	0.85 (0.72, 0.99)	0.042
Age (years)	1.03 (0.99, 1.06)	0.057	0.97 (0.93, 1.01)	0.184
Female sex	0.79 (0.41, 1.53)	0.484	0.76 (0.37, 1.59)	0.472
Employment Type				
Doctor				
Cleaning staff	0.96 (0.18, 5.24)	0.963	1.44 (0.21, 9.95)	0.712
Health worker	7.12 (1.63, 31.14)	0.009	7.92 (1.67, 37.54)	0.009
Nurse	1.00 (0.19, 5.17)	0.997	0.99 (0.19, 5.19)	0.993
Other	7.13 (1.56, 32.57)	0.011	6.21 (1.29, 29.76)	0.022
Hypertension	2.50 (1.19, 5.22)	0.015	1.68 (0.70, 3.99)	0.243
Bronchial asthma/COPD	1.40 (0.49, 3.97)	0.529		
Smoking habit	1.40 (0.49, 3.98)	0.526		
Total days of work	0.95 (0.93, 0.98)	<0.001	0.98 (0.95, 1.01)	0.267

IQR: Interquartile range; COPD: Chronic Obstructive Pulmonary Disease.

**Table 6:** Side effects in the prophylaxis group.

Side Effect	*n* (%)
No problems	208 (90.83)
Palpitation	1 (0.44)
Neurological problem	5 (2.18)
Skin problem	3 (1.31)
Visual problem	1 (0.44)
Weakness	1 (0.44)
Feverish	1 (0.44)
GI problem	7 (3.06)
Anxiety	1 (0.44)
Weakness and neurological problem	1 (0.44)

**Table 7:** ECG reports in the prophylaxis group.

Corrected QT Interval	First Time ECG			Second Time ECG		
	Overall (*n* = 229)	Control (*n* = 12)	Prophylaxis (*n* = 217)	Overall (*n* = 105)	Control (*n* = 0)	Prophylaxis (*n* = 105)
Corrected QT interval						
<450	209 (91.27%)	11 (91.67%)	198 (91.24%)	99 (94.29%)	-	99 (94.29%)
450 to 480	18 (7.86%)	1 (8.33%)	17 (7.83%)	6 (5.71%)	-	6 (5.71%)
>480	2 (0.87%)	0 (0%)	2 (0.92%)	0 (0%)	-	0 (0%)
Findings						
Atrial fibrillation, Ischemia	1 (0.44%)	0 (0%)	1 (0.44%)	0 (0%)	-	0 (0%)
Bradycardia	2 (0.87%)	0 (0%)	2 (0.87%)	1 (0.95%)	-	1 (0.95%)
Ischemia	1 (0.44%)	0 (0%)	1 (0.44%)	4 (3.81%)	-	4 (3.81%)
Ischemia, Tachycardia	1 (0.44%)	0 (0%)	1 (0.44%)	0 (0%)	-	0 (0%)
Normal	210 (91.7%)	9 (75%)	201 (92.63%)	88 (83.81%)	-	88 (83.81%)
Tachycardia	1 (0.44%)	0 (0%)	1 (0.44%)	0 (0%)	-	0 (0%)
Uncorrected prolonged QT, Irregular heart rate	1(0.44%)	0 (0%)	1 (0.44%)	0 (0%)	-	0 (0%)
Uncorrected prolonged QT	5(2.18%)	0 (0%)	5 (2.18%)	1(0.95%)	-	1 (0.95%)
Uncorrected prolonged QT, Bradycardia	0 (0%)	0 (0%)	0 (0%)	1(0.95%)	-	1 (0.95%)
Irregular heart rate	7(3.06%)	3 (25%)	4 (1.84%)	10 (9.52)	-	10 (9.52)

**Table 8:** Results of eye examination.

	Control	Prophylaxis	Both Groups	
	106	230	336	
Participants eligible for eye check-up	45 (42%)	73 (32%)	118	
Participants who completed eye check-up	7 (16%)	46 (63%)	53	
Visual acuity (VA)				
Aided (spectacles)	5 (71%)	21 (46%)	26 (49%)	0.218
Visual acuity in right eye				
6/6 (Normal)	4 (57%)	28 (61%)	32 (80%)	0.840
6/9	2 (29%)	12 (26%)	14 (26%)	0.867
6/12	0 (0%)	6 (13%)	6 (11%)	0.311
6/18	1 (14%)	0 (0%)	1 (2%)	0.010
Abnormal VA in right eye	3 (43%)	18 (39%)	21 (40%)	0.840
Visual acuity in left eye				
6/6 (Normal)	4 (57%)	31 (67%)	35 (66%)	0.604
6/9	2 (29%)	13 (28%)	15 (28%)	0.956
6/12	0 (0%)	1 (2%)	1 (2%)	0.706
6/18	1 (14%)	1 (2%)	2 (4%)	0.117
Abnormal VA in left eye	3 (43%)	15 (32%)	18 (34%)	0.566
Intra-ocular pressure, mean (sd)				
Right eye (mm Hg)	18.4 (2.5)	19.0 (3.7)	18.9 (3.6)	0.594
Left eye (mm Hg)	18.0 (2.8)	19.5 (3.7)	19.3 (3.7)	0.238
Central corneal thickness (CCT) Mean (sd)	*n* = 7	*n* = 43	*n* = 50	
Right eye (µm),	554.3 (20.1)	554.7 (26.2)	554.6 (25.3)	0.964
Left eye (µm)	561.4 (22.7)	554.4 (27.8)	555.4 (27.2)	0.483
Visual field analysis:	*n* = 7	*n* = 46	*n* = 53	
Scotoma in right eye	2 (29%)	20 (44%)	22 (44%)	0.454
Scotoma in left eye	2 (29%)	16 (35%)	18 (34%)	0.755
Over all comments:	*n* = 7	*n* = 46	*n* = 53	
Early lenticular opacity (cataract)	3 (14%)	5 (11%)	8 (15%)	0.816
Suspected glaucomatous change	1 (14%)	2 (4%)	3 (6%)	0.272
Ocular hypertension/raised intraocular pressure	0 (0%)	2 (4%)	2 (4%)	0.590
Scotoma (any eye, central/peripheral)	2 (29%)	23 (50%)	25 (47%)	0.300
Changes due to HCQ prophylaxis				
Humphrey visual field				
Superonasal visual field loss	0	0	0	
Pericentral deficits	0	0	0	
Ring scotomas	0	0	0	
Fundus photo				
Hyperfluorescence (early sign)	0	0	0	
Hypofluorescence (late sign)	0	0	0	
Optical coherence tomography	0	0	0	
Morphological changes due to HCQ retinopathy	0	0	0	

## Data Availability

All data related with this research can be made available under institutional policy. For data access, requests should be made to aahmed@icddrb.org, Head, Research Administration, icddr, b.

## Supplementary Materials

The following supporting information can be downloaded at: https://www.mdpi.com/article/10.3390/life12122047/s1, Supplementary Table S1: Decision on baseline ECG; Supplementary Table S2: Tisdale risk factor assessment score; and Supplementary Table S3: CONSORT checklist.
