# Supplementary material for: Hospital-Based Quasi-Experimental Study on Hydroxychloroquine Pre-Exposure Prophylaxis for COVID-19 in Healthcare Providers with Its Potential Side-Effects
Source: Life (Basel). 2022 Dec 7;12(12):2047. doi: 10.3390/life12122047 (PMC9786013; doi:10.3390/life12122047)
Supplement: Supplementary file 1 [file life-12-02047-s001.zip › Supplemental Table S1 & Table S2.pdf]

Supplementary Table S1: Decision on baseline ECG

**Admission QTc**	
≥480 msec-	Stop prophylaxis, do risk factor assessment (table 2) and then decide on continuation of prophylaxis
450-480 msec	Cautious prophylaxis with weekly ECG, do risk factor assessment (table 2) and decide on continuation
<450 msec	Continue prophylaxis for 7 weeks with follow up ECG on 5th week and ophthalmological examination

Supplementary Table S2: Tisdale risk factor assessment score

**Risk Factor**	**Points**
Age ≥68 y	1
Female sex	1
Taking loop diuretics	1
Serum K+ ≤3.5 mEq/L	2
Admission QTc>450 msec	2
Acute MI	2
≥2 QTc prolonging drugs	3
Sepsis	3
Heart Failure	3
One QTc prolonding drugs	3
Maximum Risk Score	21
